# Pancreatectomy with arterial resection for periampullary cancer: outcomes after planned or unplanned events in a nationwide, multicentre cohort

**DOI:** 10.1093/bjs/znac353

**Published:** 2022-10-29

**Authors:** Thomas F Stoop, Tara M Mackay, Lilly J H Brada, Erwin van der Harst, Freek Daams, Freek R van ‘t Land, Geert Kazemier, Gijs A Patijn, Hjalmar C van Santvoort, Ignace H de Hingh, Koop Bosscha, Leonard W F Seelen, Maarten W Nijkamp, Martijn W J Stommel, Mike S L Liem, Olivier R Busch, Peter-Paul L O Coene, Ronald M van Dam, Roeland F de Wilde, J Sven D Mieog, I Quintus Molenaar, Marc G Besselink, Casper H J van Eijck, the Dutch Pancreatic Cancer Group, Vincent E de Meijer, Bram Olij, Marcel den Dulk, Mark Ramaekers, Bert A Bonsing, Nynke Michiels, Bas Groot Koerkamp, Sebastiaan Festen, Fenny Wit, Daan J Lips, Werner Draaisma, Eric Manusama, Wouter te Riele

**Affiliations:** Amsterdam UMC, location University of Amsterdam, Department of Surgery, Amsterdam, The Netherlands; Cancer Center Amsterdam, Amsterdam, The Netherlands; Amsterdam UMC, location University of Amsterdam, Department of Surgery, Amsterdam, The Netherlands; Cancer Center Amsterdam, Amsterdam, The Netherlands; Department of Surgery, Regional Academic Cancer Center Utrecht, University Medical Center Utrecht/St. Antonius Hospital Nieuwegein, Utrecht & Nieuwegein, The Netherlands; Department of Surgery, Maasstad Hospital, Rotterdam, The Netherlands; Cancer Center Amsterdam, Amsterdam, The Netherlands; Amsterdam UMC, location Vrije Universiteit, Department of Surgery, Amsterdam, The Netherlands; Department of Surgery, Erasmus MC Cancer Institute, University Medical Center, Rotterdam, The Netherlands; Cancer Center Amsterdam, Amsterdam, The Netherlands; Amsterdam UMC, location Vrije Universiteit, Department of Surgery, Amsterdam, The Netherlands; Department of Surgery, Isala Clinics, Zwolle, The Netherlands; Department of Surgery, Regional Academic Cancer Center Utrecht, University Medical Center Utrecht/St. Antonius Hospital Nieuwegein, Utrecht & Nieuwegein, The Netherlands; Department of Surgery, Catharina Hospital, Eindhoven, The Netherlands; Department of Surgery, Jeroen Bosch Hospital, ‘s Hertogenbosch, The Netherlands; Department of Surgery, Regional Academic Cancer Center Utrecht, University Medical Center Utrecht/St. Antonius Hospital Nieuwegein, Utrecht & Nieuwegein, The Netherlands; Department of Surgery, University Medical Center Groningen, Groningen, The Netherlands; Department of Surgery, Radboud University Medical Center, Nijmegen, The Netherlands; Department of Surgery, Medisch Spectrum Twente, Enschede, The Netherlands; Amsterdam UMC, location University of Amsterdam, Department of Surgery, Amsterdam, The Netherlands; Cancer Center Amsterdam, Amsterdam, The Netherlands; Department of Surgery, Maasstad Hospital, Rotterdam, The Netherlands; Department of Surgery, Maastricht University Medical Center, Maastricht, The Netherlands; Department of General, Visceral and Transplant Surgery, University Hospital Aachen, Aachen, Germany; Department of Surgery, Erasmus MC Cancer Institute, University Medical Center, Rotterdam, The Netherlands; Department of Surgery, Leiden University Medical Center, Leiden, The Netherlands; Department of Surgery, Regional Academic Cancer Center Utrecht, University Medical Center Utrecht/St. Antonius Hospital Nieuwegein, Utrecht & Nieuwegein, The Netherlands; Amsterdam UMC, location University of Amsterdam, Department of Surgery, Amsterdam, The Netherlands; Cancer Center Amsterdam, Amsterdam, The Netherlands; Department of Surgery, Erasmus MC Cancer Institute, University Medical Center, Rotterdam, The Netherlands

## Introduction

Arterial resections in pancreatic surgery may be planned to obtain a radical oncological resection, or unplanned after iatrogenic injury during dissection. Most data on planned arterial resection come from single, very-high-volume centres and suggest that these resections might be feasible and even beneficial after preoperative chemotherapy in highly selected patients with pancreatic cancer^1–3^. However, real-world data on such planned and unplanned arterial resection at a nationwide level are scarce^4^. Furthermore, distinctions between planned and unplanned arterial resection are seldomly reported, even though this might have clinical implications^5,6^. The present study evaluated the incidence and surgical outcome of all planned and unplanned arterial resections for pancreatic and periampullary cancer in The Netherlands.

## Methods

The study protocol was approved by the scientific committee of the Dutch Pancreatic Cancer Group. Fifteen of 16 hospitals affiliated to the Dutch Pancreatic Cancer Group participated in this study; data were obtained from the mandatory Dutch Pancreatic Cancer Audit. Additional data were collected from local medical records.

All patients after any type of pancreatectomy with any type of concomitant arterial resection (± reconstruction) for histopathologically confirmed pancreatic or periampullary cancer (2013–2019) were included. Arterial resection comprised the hepatic artery (HA), coeliac axis (CA), and superior mesenteric artery (SMA), and was classified as ‘planned’ (i.e. performed because of arterial tumour involvement; preoperatively or intraoperatively planned) or ‘unplanned’ (i.e. performed because of iatrogenic injury). See the *Supplementary Methods* for further methodological details.

## Results

During the study period, 3868 patients underwent a pancreatectomy for pancreatic or periampullary cancer, of whom 54 (1.4 per cent) had an arterial resection. Sixty-seven per cent (*n* = 36) of procedures were planned and 31.5 per cent (*n* = 17) were unplanned (one unknown). Patients were operated on in 13 centres with an annual volume of more than 60 (three centres), 40 to 59 (five centres), or 20 to 40 (five centres) pancreatoduodenectomies. See *Fig. S1* for the inclusion flowchart and *Table 1* for the baseline patient characteristics.

**Table 1:** Characteristics of patients undergoing arterial resection

Variables	All arterial resections (*n* = 54)	Hepatic artery (*n* = 36)	Coeliac axis (*n* = 12)	Superior mesenteric artery (*n* = 6)
**Baseline**				
Age (years)	64 (58–68)	64 (56–67)	62 (57–68)	69 (65–75)
Female	31 (57)	19 (53)	8 (67)	4 (67)
Preoperative chemo(radio)therapy	18 (33)	8 (22)	9 (75)	1 (17)
ASA grade				
ȃI–II	41 (76)	25 (69)	12 (100)	4 (67)
ȃIII–IV	13 (24)	11 (31)	0 (0)	2 (33)
**Procedure**				
ȃPancreatectomy				
ȃȃPancreatoduodenectomy	35 (65)	30 (83)	0 (0)	5 (83)
ȃȃDistal pancreatectomy	12 (22)	0 (0)	11 (92)	1 (17)
ȃȃTotal pancreatectomy	7 (13)	6 (17)	1 (8)	0 (0)
Indication for arterial resection				
ȃȃPlanned	36 (67)	23 (64)†	12 (100)	1 (17)
ȃȃUnplanned	17 (32)	12 (33)	0 (0)	5 (83)
ȃȃȃDuring arterial divestment	8 (47)	6 (50)	0 (0)	2 (40)
ȃȃȃOther causes	9 (53)	6 (50)	0 (0)	3 (60)
ȃȃUnknown	1 (2)	1 (3)	0 (0)	0 (0)
ȃArterial resection with reconstruction	31 (57)	22 (61)	3 (25)	6 (100)
ȃPortomesenteric venous resection	21 (39)	13 (36)	5 (42)	3 (50)
ȃȃWedge resection	11 (52)	9 (69)	1 (20)	1 (33)
ȃȃSegment resection	10 (48)	4 (31)	4 (80)	2 (67)
ȃ(Sub)total gastrectomy	4 (7)	0 (0)	3 (25)	1 (17)
ȃColon resection	4 (7)*	1 (3)	2 (17)*	1 (17)
**Pathology**				
ȃPancreas	37 (69)	19 (53)	12 (100)	6 (100)
ȃDistal bile duct	10 (19)	10 (28)	0 (0)	0 (0)
ȃAmpulla of Vater	3 (6)	3 (8)	0 (0)	0 (0)
ȃDuodenum	4 (7)	4 (11)	0 (0)	0 (0)

Values are *n* (%) or median (interquartile range). *One missing. †One patient underwent two hepatic artery resections: one was performed because of suspected tumour involvement and the other hepatic artery was resected because of iatrogenic damage during divestment. Here, only the oncological indication is registered. For this case, this is continued throughout the whole paper.

### Surgery

The 54 arterial resections included 36 HAs (67 per cent), 13 CAs (24 per cent), and six SMAs (11 per cent). HA resections comprised the common/proper (*n* = 10), right or left (*n* = 7), aberrant (*n* = 17), and accessory (*n* = 3) HA. See *Table 1* for indications and procedural details. Any type of additional anticoagulation therapy because of arterial resection was given in 17 patients (32 per cent): 16 with arterial reconstruction and one without.

### Surgical outcome

The overall major morbidity rate was 46 per cent (25 patients) and in-hospital mortality was 11 per cent (six patients). In-hospital mortality after planned arterial resection occurred in three of 36 patients, whereas mortality after unplanned arterial resection occurred in three of 17 patients. See *Table 2* for the surgical outcomes and *Table S1* for detailed pathology results. Thirteen of 22 patients who underwent a HA resection with reconstruction experienced major morbidity and three experienced in-hospital mortality. After partial pancreatectomy (47 of 54 patients), postoperative pancreatic fistula (POPF) grade B/C occurred in 14 patients, of whom three experienced both grade B/C post-pancreatectomy haemorrhage and POPF. Subgroup analyses on patients with planned *versus* unplanned arterial resection (*Tables S2 and S3*) and on patients with pancreatic cancer (see *Tables S4 and S5*) are described in the *supplementary material*.

**Table 2:** Surgical outcome after arterial resection

Variables	All resections (n = 54)	Hepatic artery (n = 36)	Coeliac axis (n = 12)	Superior mesenteric artery (n = 6)
POPF*				
ȃGrade B	10 (21)	5 (17)	4 (36)	1 (17)
ȃGrade C	4 (9)	3 (10)	0 (0)	1 (17)
**PPH**				
ȃGrade B	4 (7)	2 (6)	0 (0)	2 (33)
ȃGrade C	4 (7)	2 (6)	1 (8)	1 (17)
ȃUnknown	2 (4)	2 (6)	0 (0)	0 (0)
**Major morbidity**	25 (46)	18 (50)	4 (33)	3 (50)
ȃRelaparotomy	6 (11)	1 (3)	2 (17)†	3 (50)
ȃȃPPH	3 (6)	1 (3)	1 (8)	1 (17)
ȃȃPOPF	0 (0)	0 (0)	0 (0)	0 (0)
ȃȃStomach ischaemia/perforation	2 (4)	0 (0)	2 (17)	0 (0)
ȃȃIntestinal ischaemia	1 (2)	0 (0)	0 (0)	1 (17)
ȃȃDelayed enteral reconstruction	1 (2)	0 (0)	0 (0)	1 (17)
ȃȃWound dehiscence	1 (2)	0 (0)	1 (8)	0 (0)
ȃSingle organ failure	4 (7)‡	4 (11)‡	0 (0)	0 (0)
ȃMultiorgan failure	5 (9)‡	1 (3)‡	1 (8)	3 (50)
ȃMCU/ICU admission	8 (15)	4 (11)	1 (8)	3 (50)
In-hospital mortality	6 (11)	4 (11)	0 (0)	2 (33)
Hospital stay (days)	17 (10–26)	17 (13–26)	14 (8–27)	12 (7–79)
Readmission¶	8 (17)§	6 (19)‡	1 (8)‡	1 (25)

Values are *n* (%) or median (interquartile range). *Patients who underwent a total pancreatectomy were excluded. †One patient underwent three relaparotomies for postpancreatectomy haemorrhage (PPH), stomach ischaemia/perforation, and wound dehiscence, respectively. ‡One missing. §Two missing . ¶Patients who died during admission (i.e. in-hospital mortality) were excluded for the nominator. POPF, postoperative pancreatic fistula; MCU, medium care unit; ICU, intensive care unit.

## Discussion

This nationwide retrospective multicentre study found that pancreatectomy combined with major arterial resection for pancreatic and periampullary cancer in The Netherlands is very rare (less than 1.5 per cent of all pancreatic resections) with high in-hospital major morbidity and mortality after HA or SMA resection. One-third of arterial resections was unplanned, and mortality was twice as high than after planned arterial resection (17.6 per cent *versus* 8.3 per cent).

Only a few single-centre studies have addressed this topic. A high-volume single-centre retrospective study reported a 0.91 per cent incidence rate of unplanned arterial resection in 1535 pancreatectomies with non-significantly higher mortality (14 per cent *versus* 5 per cent) rate compared with planned arterial resection^5^. A systematic review on unplanned HA resections confirmed the high mortality^6^. In the current era, with improved induction chemotherapy, planned arterial resections are becoming increasingly accepted when performed in highly selected patients with borderline resectable (BRPC) and locally advanced pancreatic cancer (LAPC), performed in experienced, high-volume centres^1–3^. This trend is confirmed by recent literature suggesting improved surgical safety and improved survival^7^.

HA resections in patients with pancreatic and periampullary cancer comprises a wide spectrum of procedure types whereby different HA branches can be involved and resected, eventually combined with various reconstruction types^8^. Miyazaki *et al*. reported a 52 per cent major morbidity rate without mortality after common HA resections (20 of 21 without reconstruction)^9^, whereas some experienced centres presented mortality rates up to 13–17 per cent^2,3^. These latter series included HA reconstructions with end-to-end anastomosis, transpositions and/or interposition grafts. In the current nationwide study, major morbidity (59 per cent) and mortality (14 per cent) rates after HA resections with any reconstruction seem comparable.

In contrast to the relatively large number of centres that performed HA resection(s) in this series (13 centres), (modified) Appleby procedures were performed in only five centres. No in-hospital mortality occurred (excluding one patient who underwent both CA and SMA resection), probably as result of patient selection. This is in line with the high-volume experience in Johns Hopkins, which reported a 19 per cent major morbidity rate without 30-day mortality^10^.

An SMA resection is the most challenging and is associated with very poor outcomes^11^, as confirmed by our results (i.e. 50 per cent major morbidity and 33 per cent mortality rates). Nevertheless, three single-centre, high-volume series with 79 SMA resections presented an acceptable mortality rate of up to 7 per cent^1–3^.

In general, the overall major morbidity and mortality in the current cohort of patients undergoing arterial resection substantially exceed the internationally established benchmarks for portomesenteric venous resection in pancreatic surgery (i.e. 28 per cent or less major morbidity and 4 per cent or less mortality)^12^. This underlines the fact that arterial resections concern a different entity, with higher risks. In particular, mortality after arterial resection because of iatrogenic damage was high. However, many confounders may explain this high mortality rate, such as the need and type of vascular reconstruction. To avoid life-threatening erosive bleeding after arterial resection by POPF, some advocate for total pancreatectomy^13^, which is debated by others^3^. This decision should be made on a case-by-case basis, balancing POPF risk factors and the metabolic insufficiencies^14^.

In a retrospective French multicentre series, arterial resection was performed in 2 per cent of all pancreatic cancer resections, and was associated with a 30-day mortality rate of 8 per cent^15^. An analysis in the nationwide American College of Surgeons database identified pancreatoduodenectomy with arterial resection and reconstruction as a predictor for increased morbidity and mortality^4^. Several international high-volume centres have provided insight into their learning curves in performing major arterial resections in pancreatic cancer surgery. The Mayo Clinics identified increasing experience as an independent prognostic factor for reduced mortality^2^. This was confirmed by a retrospective, high-volume, single-centre study that demonstrated a learning curve of 15 arterial resections for already highly experienced pancreatic surgeons to reduce in-hospital mortality^3^.

Besides technical expertise, pre- and intraoperative oncological and surgical selection based on anatomical, biological, and conditional parameters is key to further reducing the chance of futile surgery^16^. Napoli *et al*. developed a nomogram to predict survival for patients with LAPC requiring arterial resection with a median overall survival of 14, 24, and 31 months in high-, intermediate-, and low-risk patients, respecitvely^17^. Additionally, the presence of a halo or string sign around an artery could help in the selection of patients for arterial divestment or resection^18,19^. Intraoperative ultrasonography and frozen section biopsies could further distinguish between vital tumour and fibrotic tissue after preoperative chemo(radio)therapy^20^.

The present study should be considered in the light of several limitations. Firstly, the current sample size was too small to investigate potential predictors for morbidity and mortality. Considering the small sample size and heterogeneity in procedure types and indications, comparing surgical outcomes between centres from different volume categories was not possible. Because of the retrospective nature of the study, detailed data on postoperative anticoagulant therapy was considered unreliable and therefore was not described. Owing to the rarity of arterial resection in pancreatic surgery, indications for and the type of additional postoperative anticoagulant therapy are unstandardized. The efficacy of (additional) anticoagulant regimens should be investigated in future studies. Despite these limitations, the present study provides a realistic and unique insight in the serious surgical complications of these rarely performed procedures on a nationwide level. The oncological benefit of arterial resection for BRPC–LAPC needs to be further investigated with proper comparative analyses. Furthermore, although unplanned arterial resections are extremely rare, they should be prevented, given the high associated mortality rate.

## Collaborators

Vincent E. de Meijer, Department of Surgery, University Medical Center Groningen, Groningen, The Netherlands; Bram Olij, Department of Surgery, Maastricht University Medical Center, Maastricht, The Netherlands; Marcel den Dulk, Department of Surgery, Maastricht University Medical Center, Maastricht, The Netherlands, and Department of General, Visceral and Transplant Surgery, University Hospital Aachen, Aachen, Germany; Mark Ramaekers, Department of Surgery, Catharina Hospital, Eindhoven, The Netherlands; Bert A. Bonsing, Department of Surgery, Leiden University Medical Center, Leiden, The Netherlands; Nynke Michiels, Department of Surgery, Leiden University Medical Center, Leiden, The Netherlands; Bas Groot Koerkamp, Department of Surgery, ErasmusMC Cancer Institute, University Medical Center, Rotterdam, The Netherlands; Sebastiaan Festen, Department of Surgery, OLVG, Amsterdam, The Netherlands; Fenny Wit, Department of Surgery, Tjongerschans Hospital, Heerenveen, The Netherlands; Daan J. Lips, Department of Surgery, Medisch Spectrum Twente, Enschede, The Netherlands; Werner Draaisma, Department of Surgery, Jeroen Bosch Hospital, 's Hertogenbosch, The Netherlands; Eric Manusama, Department of Surgery, Medical Centre Leeuwarden, Leeuwarden, The Netherlands; Wouter te Riele, Department of Surgery, Regional Academic Cancer Center Utrecht, University Medical Center Utrecht/St. Antonius Hospital Nieuwegein, Utrecht & Nieuwegein, The Netherlands.

## Supplementary Material

znac353_Supplementary_Data

## Data Availability

The full manuscript data has been read and approved by all authors and collaborators.
