# Supplementary material for: Pancreatectomy with arterial resection for periampullary cancer: outcomes after planned or unplanned events in a nationwide, multicentre cohort
Source: Br J Surg. 2022 Oct 29;110(6):638–42. doi: 10.1093/bjs/znac353 (PMC10364546; doi:10.1093/bjs/znac353)
Supplement: znac353_Supplementary_Data [file znac353_supplementary_data.docx]

**Pancreatectomy with arterial resection for periampullary cancer: outcomes after planned or unplanned events in a nationwide, multicenter cohort**

Thomas F. Stoop^1,2^*; Tara M. Mackay^1,2^*; Lilly J.H. Brada^3^; Erwin van der Harst^4^; Freek Daams^2,5^; Freek van ’t Land^6^; Geert Kazemier^2,5^; Gijs A. Patijn^7^; Hjalmar C. van Santvoort^3^; Ignace H. de Hingh^8^; Koop Bosscha^9^; Leonard W.F. Seelen^3^; Maarten W. Nijkamp^10^; Martijn W.J. Stommel^11^; Mike Liem^12^; Olivier R. Busch^1,2^; Peter-Paul L.O. Coene^4^; Ronald M. van Dam^13,14^; Roeland F. de Wilde^6^; J. Sven D. Mieog^15^; I. Quintus Molenaar^3^; Marc G. Besselink^1,2^; Casper H.J. van Eijck^6^ for the Dutch Pancreatic Cancer Group

^1^Amsterdam UMC, location University of Amsterdam, Department of Surgery, Amsterdam, The Netherlands;

^2^Cancer Center Amsterdam, The Netherlands;

^3^Department of Surgery, Regional Academic Cancer Center Utrecht, University Medical Center Utrecht / St. Antonius Hospital Nieuwegein, The Netherlands;

^4^Department of Surgery, Maasstad Hospital, Rotterdam, The Netherlands;

^5^Amsterdam UMC, location Vrije Universiteit, Department of Surgery, The Netherlands

^6^Department of Surgery, ErasmusMC Cancer Institute, University Medical Center, Rotterdam, The Netherlands;

^7^Department of Surgery, Isala Clinics, Zwolle, The Netherlands;

^8^Department of Surgery, Catharina Hospital, Eindhoven, The Netherlands;

^9^Department of Surgery, Jeroen Bosch Hospital, ‘s Hertogenbosch, The Netherlands;

^10^Department of Surgery, University Medical Center Groningen, Groningen, The Netherlands;

^11^Department of Surgery, Radboud University Medical Center, Nijmegen, The Netherlands;

^12^Department of Surgery, Medisch Spectrum Twente, Enschede, The Netherlands;

^13^Department of Surgery, Maastricht University Medical Center, Maastricht, The Netherlands;

^14^Department of General, Visceral and Transplant Surgery, University Hospital Aachen, Germany

^15^Department of Surgery, Leiden University Medical Center, Leiden, The Netherlands;

* shared first author.

**Corresponding author**

Casper H. J. van Eijck, MD, PhD, Professor of Surgery

Department of Surgery, P.O. box 2040

Erasmus MC, 3000 CA Rotterdam, the Netherlands

E-mail address: [c.vaneijck@erasmusmc.nl](mailto:c.vaneijck@erasmusmc.nl)

ORCID ID: 0000-0002-9511-2157

Twitter: @Caspervaneijck

**Supplementary Materials – Index**

| **Supplementary Methods** |  |
| --- | --- |
| Methodology | *pag. 3* |
| **Supplementary Figures and Tables** |  |
| Figure S1. Flow chart inclusion | *pag. 4* |
| Table S1. Pathology  Table S2. Baseline characteristics – Planned & unplanned arterial resections  Table S3. Surgical outcome – Planned & unplanned arterial resections  Table S4**.** Pancreatic cancer – Baseline characteristics  Table S5. Pancreatic cancer – Surgical outcome | *pag. 5*  *pag. 6*  *pag. 8*  *pag. 9*  *pag. 10* |
| **References** | *pag. 11* |
|  |  |

**Supplementary Methods**

Methodology

Patients’ preoperative health status was expressed with the American Society of Anesthesiologists Physical Status classification.

Pancreatic resections and its extent were defined, according to the International Study Group on Pancreatic Surgery (ISGPS).^1^ Patients who underwent multiple major AR were placed in only one of the groups (i.e. hepatic artery, celiac axis, or superior mesenteric artery) when evaluating surgical outcomes. That group was chosen, based on the type of AR that presumably is associated with the worst surgical outcome; superior mesenteric artery, celiac axis, and hepatic artery (in order from poor to better).

Major morbidity was defined as Clavien Dindo grade ≥IIIa.^2^ Postpancreatectomy hemorrhage and postoperative pancreatic fistula (POPF) were classified following the ISGPS definition.^3,4^ The 2005 ISGPS POPF definition was converted to the 2016 definition in patients who were operated before the use of the 2016 definition. Readmission was defined as hospital admission within 30 days after discharge.

Pancreatic and periampullary (i.e. duodenal, distal bile duct, and ampulla of Vater) cancers (i.e. adenocarcinomas) were defined using the World Health Organization definitions^5^ whereby the Royal College of Pathologist definition was used to classify radicality.^6^

All data are presented descriptively and no statistical comparative tests were performed because of the small sample sizes and heterogeneity.

**Supplementary Figures and Tables**

**FIGURE S1.** Inclusion flow diagram

Pancreatic resection for pancreatic & periampullary cancer

*n*=3868

Reason of exclusion:

(1) No arterial resection, *n*=3769

Reason of exclusion:

(1) Other type of arterial resection(s), *n*=30

- Splenic artery, *n*=21
- Major colic artery, *n*=4
- Left gastric artery, *n*=2
- Branch(es) SMA, *n*=2
- Others, *n*=4
- Unknown, *n*=3

(2) No arterial resection, *n*=9

(3) Other postoperative histological diagnosis, *n*=3:

- Pancreatic metastases, *n*=3

(4) Other type of primary procedure, *n*=1

- Liver transplantation, *n*=1

Pancreatectomy with arterial resection for pancreatic & periampullary cancer

*n*=97

Pancreatectomy with arterial resection for pancreatic & periampullary cancer

*n*=54

*n,* number of patients; *SMA*, superior mesenteric artery.

| **TABLE S1.** Pathology | | | | |
| --- | --- | --- | --- | --- |
| **Variables** | **Pancreas** | **Distal bile duct** | **Ampulla of Vater** | **Duodenum** |
| **Patients, *n* (%)** | **37 (68^.^5)** | **10 (18^.^5)** | **3 (5^.^6)** | **4 (7^.^4)** |
| Tumor size, median (IQR) (mm)  Missing, *n* (%) | 28 (20-40)  3 (8^.^1) | 24 (20-27)  1 (10^.^0) | 15 (15-NA)  0 (0) | 30 (18-54)  0 (0) |
| Lymph nodes  Total, median (IQR)  Positive resected, median (IQR)  Missing | 14 (12-22)  1 (0-3)  2 (5^.^4) | 14 (9-22)  2 (1-5)  0 (0) | 11 (10-NA)  0 (0-NA)  0 (0) | 13 (11-15)  5 (2-9)  (0) |
| Metastatic disease, *n* (%)  M0  M1  Unknown  Missing | 32 (86^.^5)  3 (8^.^1)  1 (2^.^7)  1 (2^.^7) | 9 (90^.^0)  1 (10^.^0)  0 (0)  0 (0) | 3 (100)  0 (0)  0 (0)  0 (0) | 2 (50^.^0)  2 (50^.^0)  0 (0)  0 (0) |
| Differentiation, *n* (%)  Well  Moderate  Poor  Missing / unknown | 2 (5^.^4)  18 (48^.^6)  13 (35^.^1)  4 (10^.^8) | 2 (20^.^0)  4 (40^.^0)  3 (30^.^0)  1 (10^.^0) | 1 (33^.^3)  2 (66^.^7)  0 (0)  0 (0) | 3 (75^.^0)  0 (0)  1 (25^.^0)  0 (0) |
| Radicality*, *n* (%)  R0  R1  Missing | 14 (37^.^8)  23 (62^.^2)  0 (0) | 1 (10^.^0)  9 (90^.^0)  0 (0) | 3 (100)  0 (0)  0 (0) | 4 (100)  0 (0)  0 (0) |
| *n*, number of patients; *mm,* millimetres; *IQR,* interquartile range; *NA*, not available; *, none of the patients was considered as R2 by the surgeon. | | | | |

| **TABLE S2.** Baseline characteristics – Planned & unplanned arterial resections | | | |
| --- | --- | --- | --- |
| **Variables** | **Planned** | **Unplanned** | |
| **Patients, *n* (%)** | **36 (66^.^7)** | **17 (31^.^5)** | |
| **BASELINE** | | | |
| Age, median (IQR) (years) | 64 (57-68) | 65 (61-73) | |
| Female, *n* (%) | 21 (58^.^3) | 9 (52^.^9) | |
| Preoperative chemo(radio)therapy, *n* (%) | 16 (44^.^4) | 1 (5^.^9) | |
| ASA PS, *n* (%)  I-II  III-IV | 29 (80^.^6)  7 (19^.^4) | 12 (70^.^6)  5 (29^.^4) | |
| **PROCEDURE** | | | |
| Pancreatectomy, *n* (%)  Pancreatoduodenectomy  Distal pancreatectomy  Total pancreatectomy | 19 (52^.^8)  12 (33^.^3)  5 (13^.^9) | | 16 (94^.^1)  0 (0)  1 (5^.^9) |
| Arterial resection, *n* (%)  Hepatic artery  *Common / proper hepatic artery*  *Right / left hepatic artery*  *Aberrant hepatic artery*  *Accessory hepatic artery*  *With reconstruction*  Celiac axis  *With reconstruction*  Superior mesenteric artery | 23 (63.9)  6 (26^.^1)  3 (13^.^0)  13 (56^.^5)  2 (8^.^7)  15 (65^.^2)  13 (36^.^1)  3 (23^.^1)  1 (2^.^8) | | 12 (70^.^6)  3 (25^.^0)  4 (33^.^3)  4 (33^.^3)  1 (8^.^3)  6 (50^.^0)  0 (0)  0 (0)  5 (29^.^4) |
| Portomesenteric venous resection, *n* (%)  Wedge resection  Segment resection  (Sub)total gastrectomy, *n* (%)  Colon resection, *n* (%) | 13 (36^.^1)  5 (38^.^5)  8 (61^.^5)  4 (11^.^1)  2 (5^.^6)* | | 7 (41^.^2)  5 (71^.^4)  2 (28^.^6)  0 (0)  2 (11^.^8) |
| **PATHOLOGY** | | | |
| Pancreas  Distal bile duct  Ampulla of Vater  Duodenum | 25 (69^.^4)  8 (22^.^2)  1 (2^.^8)  2 (5^.^6) | | 11 (64^.^7)  2 (11^.^8)  2 (11^.^8)  2 (11^.^8) |
| *n*, number of patients; *, *n*=1 missing; #, one patient underwent two hepatic artery resections of whom one was performed because of suspected tumor involvement while the other hepatic artery was resected because of iatrogenic damage during divestment. Here, the patient is registered in the group of intentional arterial resections. | | | |

| **TABLE S3.**  Surgical outcome – Planned & unplanned arterial resections | | |
| --- | --- | --- |
| **Variables** | **Planned** | **Unplanned** |
| **Patients, *n* (%)** | **36 (66^.^7)** | **17 (31^.^5)** |
| POPF, *n* (%)^#^  Grade B  Grade C | 7 (22^.^6)  2 (6^.^5) | 3 (18^.^8)  2 (12^.^5) |
| PPH, *n* (%)  Grade B  Grade C  Unknown | 3 (8^.^3)  2 (5^.^6)  0 (0) | 1 (5^.^9)  2 (11^.^8)  2 (11^.^8) |
| Major morbidity, *n* (%)  Relaparotomy  Single organ failure  Multi organ failure  MCU / ICU admission | 17 (47^.^2)  4 (11^.^1)  3 (8^.^3)*  3 (8^.^3)*  6 (16^.^7) | 7 (41^.^2)  2 (11^.^8)  1 (5^.^9)  2 (11^.^8)  2 (11^.^8) |
| In-hospital mortality, *n* (%) | 3 (8^.^3) | 3 (17^.^6) |
| Hospital stay, median (IQR) (days) | 17 (9-27) | 18 (14-26) |
| Readmission, *n* (%)^⌘^ | 6 (18^.^2)** | 2 (11^.^8) |
| *n*, number of patients; *PPH*, postpancreatectomy hemorrhage;  *POPF,* postoperative pancreatic fistula; *MCU*, medium care unit; *ICU*, intensive care unit; #, patients who underwent a total pancreatectomy were excluded; *, missing *n*=1; **, missing *n*=2; ⌘, patients who died during admission (i.e. in-hospital mortality) are excluded for the nominator. | | |

| **TABLE S4.** Pancreatic cancer – Baseline characteristics | | | |
| --- | --- | --- | --- |
| **Variables** | **Overall** | **Upfront surgery** | **Preoperative therapy** |
| **Patients, *n* (%)** | **37 (100)** | **20 (54^.^1)** | **17 (45^.^9)** |
| **BASELINE** | | | |
| Age, median (IQR) (years) | 64 (58-68) | 65 (57-72) | 64 (58-66) |
| Female, *n* (%) | 22 (59^.^5) | 11 (55^.^0) | 11 (64^.^7) |
| ASA-PS, *n* (%)  I-II  III-IV | 27 (73^.^0)  10 (27^.^0) | 13 (65^.^0)  7 (35^.^0) | 14 (82^.^4)  3 (17^.^6) |
| **PROCEDURE** | | | |
| Pancreatectomy, *n* (%)  Pancreatoduodenectomy  Distal pancreatectomy  Total pancreatectomy | 19 (51^.^4)  12 (32^.^4)  6 (16^.^2) | 15 (75^.^0)  3 (15^.^0)  2 (10^.^0) | 4 (23^.^5)  9 (52^.^9)  4 (23^.^5) |
| Hepatic artery, *n* (%)  Common / proper hepatic artery  Right / left hepatic artery  Aberrant hepatic artery  Accessory hepatic artery  Celiac axis, *n* (%)  Superior mesenteric artery, *n* (%)  Portomesenteric venous resection, *n* (%)  (Sub)total gastrectomy, *n* (%)  Colon resection, *n* (%) | 19 (51^.^4)  8 (42^.^1)  2 (10^.^5)  6 (31^.^6)  3 (15^.^8)  13 (35^.^1)  6 (16^.^2)  19 (51^.^4)  4 (10^.^8)  3 (8^.^3)* | 12 (60^.^0)  4 (33^.^3)  2 (16^.^7)  4 (33^.^3)  2 (16^.^7)  3 (15^.^0)  5 (25^.^0)  10 (50^.^0)  0 (0)  1 (5^.^0)* | 7 (41^.^2)  4 (57^.^1)  0 (0)  2 (28^.^6)  1 (14^.^3)  10 (58^.^8)  1 (5^.^9)  9 (52^.^9)  4 (23^.^5)  2 (11^.^8) |
| *n*, number of patients; *IQR,* interquartile range; *ASA-PS,* American Society of Anesthesiologists Performance Status; *, missing *n*=1. | | | |

| **TABLE S5.** Pancreatic cancer – Surgical outcome | | | |
| --- | --- | --- | --- |
| **Variables** | **Overall** | **Upfront surgery** | **Preoperative therapy** |
|  |  |  |  |
| **Patients, *n* (%)** | **37 (100)** | **20 (54^.^1)** | **17 (45^.^9)** |
| POPF, *n* (%)^#^  Grade B  Grade C | 6 (19^.^4)  2 (6^.^5) | 3 (16^.^7)  2 (11^.^1) | 3 (23^.^1)  0 (0) |
| PPH, *n* (%)  Grade B  Grade C | 2 (5^.^4)  4 (10^.^8) | 1 (5^.^0)  3 (15^.^0) | 1 (5^.^9)  1 (5^.^9) |
| Major morbidity, *n* (%)  Relaparotomy  Single organ failure  Multi organ failure  MCU / ICU admission | 15 (40^.^5)  5 (13^.^5)  1 (2^.^7)*  5 (13^.^5)*  6 (16^.^2) | 8 (40^.^0)  3 (15^.^0)  1 (5^.^9)  4 (20^.^0)  5 (25^.^0) | 7 (41^.^2)  2 (11^.^8)  0 (0)  1 (5^.^9)*  1 (5^.^9) |
| In-hospital mortality, *n* (%) | 4 (10^.^8) | 3 (15^.^0) | 1 (5^.^9) |
| Hospital stay, median (IQR) (days) | 14 (9-29) | 20 (12-35) | 9 (8-22) |
| Readmission, *n* (%)^⌘^ | 2 (6^.^1)** | 2 (11^.^8)* | 0 (0)* |
| *n*, number of patients; *PPH,* postpancreatectomy hemorrhage; *POPF,* postoperative pancreatic fistula; *MCU*, medium care unit; *ICU*, intensive care unit; #, patients who underwent a total pancreatectomy were excluded; *, missing *n*=1; **, missing *n*=2; ⌘, patients who died during admission (i.e. in-hospital mortality) are excluded for the nominator. | | | |

**References**

1. Hartwig W, Vollmer CM, Fingerhut A, et al. Extended pancreatectomy in pancreatic ductal adenocarcinoma: definition and consensus of the International Study Group for Pancreatic Surgery (ISGPS). *Surgery* 2014;**156**:1-14.

2. Clavien PA, Barkun, J, de Oliveira, ML, et al. The Clavien-Dindo classification of surgical complications: five-year experience. *Ann Surg* 2009;**250**:187-196.

3. Wente MN, Veit JA, Bassi C, et al. Postpancreatectomy hemorrhage (PPH): an International Study Group of Pancreatic Surgery (ISGPS) definition. *Surgery* 2007;**142**:20-25.

4. Bassi C, Marchegiani G, Dervenis C, et al. The 2016 update of the International Study Group (ISGPS) definition and grading of postoperative pancreatic fistula: 11 years after. *Surgery* 2017*;***161**:584-591.

5. Bosman FT, Carneiro F, Hruban RH, Triese ND. WHO Classification of Tumors of the Digestive System, 4^th^ edn. *Lyon: IARC Press* 2010.

6. Campbell F, Foulis A, Verbeke C. Dataset for the histopathological reporting of carcinomas of the pancreas, ampulla of Vater and common bile duct. *The Royal College of Pathologists.* 2010.
